# Effects of a health coaching program based on Cox’s interaction model in older adults with diabetes mellitus in Korea: a quasi-experimental study

**DOI:** 10.7586/jkbns.25.056

**Published:** 2025-11-28

**Authors:** Hye Seung Kang

**Affiliations:** Department of Nursing, Saekyung University, Yeongwol-gun, Gangwon-do, Korea

**Keywords:** Aged, Diabetes mellitus, Glycated hemoglobin, Nurse-patient relations, Self care

## Abstract

**Purpose:**

This study aimed to evaluate the effects of a health coaching program based on Cox’s interaction model in adults aged 65 years or older with diabetes mellitus.

**Methods:**

A quasi-experimental design with a non-equivalent control group and pretest–posttest measures was employed. Fifty-nine participants were assigned to either the experimental group (n = 30) or the control group (n = 29). The 8-week intervention included 16 sessions of group coaching, health education, stress management, and health monitoring. Outcome measures included physiological indicators (glycated hemoglobin, fasting blood glucose, total cholesterol, high-density lipoprotein cholesterol, and blood pressure), self-care behaviors, diabetes distress, and health conservation.

**Results:**

The experimental group demonstrated significant reductions in glycated hemoglobin (t = 4.59, *p* < .001), fasting blood glucose (t = 6.66, *p* < .001), total cholesterol (t = 2.90, *p* = .005), systolic blood pressure (t = 2.32, *p* = .024), and diastolic blood pressure (t = 2.94, *p* = .005) versus the control group. Psychosocial outcomes also improved, with increased self-care behaviors (t = 4.04, *p* < .001) and health conservation (t = 3.20, *p* = .002), along with decreased diabetes distress (t = 3.80, *p* < .001).

**Conclusion:**

The health coaching program based on Cox’s interaction model significantly improved both physiological and psychosocial outcomes in older adults with diabetes. These findings support the model’s applicability for designing patient-centered nursing interventions to promote self-management and enhance community-based chronic care.

## INTRODUCTION

### 1. Background

In South Korea, the prevalence of diabetes among older adults has continued to rise alongside rapid population aging. Recent reports indicate that approximately 29.6% of individuals aged 65 years and older have diabetes, meaning that nearly three in ten older adults are affected. Moreover, about 70% of these individuals have one or more comorbid conditions [1]. Diabetes is associated with serious complications such as cardiovascular and cerebrovascular diseases, which contribute to premature mortality, functional decline, and diminished quality of life [2]. Therefore, there is an urgent need for more individualized and comprehensive strategies for managing older adults with diabetes [3].

The primary goals of diabetes management are to achieve optimal glycemic control and prevent complications [4]. This requires maintaining appropriate levels of key physiological indicators, including glycated hemoglobin (HbA1c), fasting blood glucose (FBG), total cholesterol (TC), high-density lipoprotein cholesterol (HDL-C), and blood pressure [5]. In addition to pharmacological treatment, consistent self-care behaviors—such as dietary regulation and regular physical activity—are essential for effective management [6].

Older adults with diabetes often face difficulties in self-management due to multiple comorbidities and age-related syndromes, including depression, cognitive impairment, visual deficits, and polypharmacy [7]. They also experience diabetes-related distress caused by disease complications, the psychological burden of glycemic control, treatment costs, social isolation, and low self-efficacy [8,9]. For this population, health conservation, defined as maintaining the highest possible level of functioning while considering life expectancy and quality of life, is a critical goal [10]. Effective self-management not only improves glycemic control and reduces complications but also supports overall health maintenance [11]. Hence, individualized and theory-based interventions that address the unique needs of older adults are essential [12].

Health coaching has emerged as an effective strategy to enhance self-management in individuals with chronic conditions by fostering intrinsic motivation and promoting health-promoting behaviors through interactive engagement [13]. It facilitates goal setting and achievement, making it particularly suitable for chronic disease management. Previous studies have demonstrated that health coaching programs can improve physiological outcomes, reduce diabetes distress, and enhance self-efficacy and quality of life among patients with diabetes [14,15].

Given that health coaching is fundamentally based on interaction, Cox’s Interaction Model of Client Health Behavior provides a robust theoretical foundation for its application [16]. The model emphasizes systematic and sustained interaction between clients and healthcare professionals, thereby enhancing motivation and encouraging health-promoting behaviors to maintain and improve health [17]. Nursing interventions grounded in Cox’s model have shown positive effects on physiological outcomes, self-care behaviors, and quality of life across diverse populations, including community-dwelling older adults and patients with chronic illnesses [18–21].

Building on this evidence, the present study developed and applied a health coaching program based on Cox’s Interaction Model of Client Health Behavior to community-dwelling older adults with diabetes. This theory-based and individualized approach aimed to improve glycemic control, strengthen self-care capacity, reduce diabetes distress, and promote long-term health conservation in the rapidly growing elderly diabetic population.

### 2. Study aim

The purpose of this study was to develop a health coaching program based on Cox’s Interaction Model of Client Health Behavior and to evaluate its effects on physiological indicators, self-care behaviors, diabetes distress, and health conservation among older adults with diabetes.

### 3. Hypothesis

The hypotheses of this study were as follows:

**Hypothesis 1:** The experimental group that received the health coaching intervention will show greater improvement in physiological indicators than the control group that did not receive the intervention.

**Sub-hypothesis 1-1:** HbA1c levels will decrease more in the experimental group than in the control group.

**Sub-hypothesis 1-2:** FBG levels will decrease more in the experimental group than in the control group.

**Sub-hypothesis 1-3:** TC levels will decrease more in the experimental group than in the control group.

**Sub-hypothesis 1-4:** HDL-C levels will increase more in the experimental group than in the control group.

**Sub-hypothesis 1-5:** Systolic blood pressure will decrease more in the experimental group than in the control group.

**Sub-hypothesis 1-6:** Diastolic blood pressure will decrease more in the experimental group than in the control group.

**Hypothesis 2.** The experimental group that received the health coaching intervention will have a greater increase in self-care behavior scores than the control group.

**Hypothesis 3.** The experimental group that received the health coaching intervention will have a greater decrease in diabetes distress scores than the control group.

**Hypothesis 4.** The experimental group that received the health coaching intervention will have a greater increase in health conservation scores than the control group.

## METHODS

### 1. Study design

This quasi-experimental study employed a non-equivalent control group pretest-posttest design to evaluate the effects of a health coaching program based on Cox’s interaction model in older adults with diabetes.

### 2. Participants

The sample size was calculated using G*Power 3.1.9 [22] for an independent t-test, with an effect size of .80, a significance level of .05, and a statistical power of .90. The effect size was based on a previous study by Ko and Lee [23] that examined the effects of a lifestyle coaching intervention on HbA1c. A minimum of 28 participants per group was required, and considering a 10% potential dropout rate, 31 participants were recruited for each group, yielding a total sample size of 62.

Participants in the experimental group were recruited from Gyeongsan City Senior Welfare Center, and those in the control group from Yeongcheon City Comprehensive Social Welfare Center, selected for their comparable regional and environmental characteristics to minimize contamination. The control group did not receive any intervention during the study period; however, they underwent all scheduled assessments, including blood tests and questionnaires, at the same time points as the experimental group. This ensured that any differences in outcomes could be attributed solely to the health coaching program.

**Inclusion criteria** were: (1) age ≥ 65 years, (2) diagnosed with diabetes mellitus for at least 1 year, (3) currently taking oral hypoglycemic agents, (4) Mini-Mental State Examination-Korean version (MMSE-K) score ≥ 24, and (5) provided written informed consent.

**Exclusion criteria** included: diagnosis of cardiovascular disease, stroke, neurological or psychiatric disorders; current insulin therapy; or other serious comorbidities.

**Figure 1.**
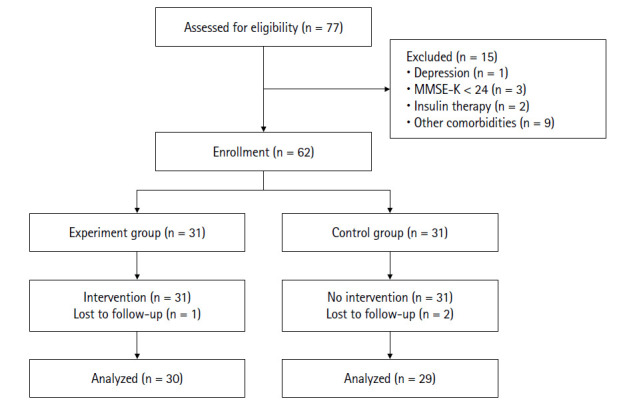
Flow chart of study participants. MMSE-K = Mini-Mental State Examination-Korean version.

Of the 77 older adults who enrolled in the study, 15 were excluded: 4 due to cardiovascular disease, 1 due to a psychiatric disorder, 3 with MMSE-K scores below 24, 2 undergoing insulin therapy, and 5 with other medical conditions. Consequently, 62 participants were enrolled, with 31 assigned to the experimental group and 31 to the control group. During the intervention period, 1 participant in the experimental group withdrew due to personal reasons (travel to visit family in another region), and 2 in the control group failed to complete the post-test. The final analysis included 59 participants: 30 in the experimental group and 29 in the control group. The flow of participants through each stage of the study is presented in Figure 1.

### 3. Instruments

#### 1) Physiological indicators: HbA1c, FBG, TC, HDL-C, and Blood pressure

Participants fasted for at least 8 hours before blood collection, and compliance was verified on the test day. Samples were centrifuged, refrigerated, and sent to the Green Cross Clinical Laboratory for analysis. Pre- and post-intervention tests were conducted at the same site and time of day to ensure consistency. As HbA1c reflects average blood glucose over 2~3 months, it was measured 4 weeks after program completion. HbA1c was analyzed using High performance liquid chromatography with the Variant II system (Bio-Rad Laboratories, Hercules, CA, USA), and FBG, TC, and HDL-C were measured by enzymatic colorimetric assay with the Modular Analytics PE system (Roche Diagnostics, Mannheim, Germany).

Blood pressure was measured using an automatic sphygmomanometer (CITIZEN Systems Co., Ltd., Tokyo, Japan). Participants rested in a seated or supine position for at least 10 minutes prior to measurement. The cuff was placed on the upper arm at heart level, with its lower edge positioned 2 cm above the brachial artery. Two measurements were taken at 5-minute intervals, and the average was used for analysis.

#### 2) Self-care behaviors

Self-care behaviors were measured using a validated instrument developed by Kim [24], consisting of 20 items across five domains: general health management (5 items), dietary management (7 items), physical activity (2 items), medication adherence (3 items), and blood glucose testing (3 items). Each item was rated on a 5-point Likert scale from 1 (“never”) to 5 (“always”), with total scores ranging from 20 to 100. Higher scores reflected better self-care performance. Cronbach’s α was .85 in the original study and .82 in the current study.

#### 3) Diabetes distress

Diabetes distress was assessed using the Diabetes Distress Scale developed by Polonsky et al. [25], and translated into Korean by Choi [26], with permission. The instrument consists of 17 items across four subdomains: physician-related distress (4 items), regimen-related distress (5 items), emotional burden (5 items), and interpersonal distress (3 items). Each item was rated on a 5-point Likert scale, with total scores ranging from 17 to 85; higher scores indicate greater diabetes-related distress. Cronbach’s α was .87 in the original version, .86 in the Korean version, and .80 in the present study.

#### 4) Health conservation

Health conservation was assessed using the instrument developed by Sung [10] for institutionalized older adults, with permission. To adapt the scale for community-dwelling older adults, content validity was reviewed by two nursing professors specializing in gerontology and four certified gerontological nurses. The instrument comprises 37 items across four subdomains: structural integrity (8 items), energy conservation (8 items), personal integrity (14 items), and social integrity (7 items). Each item was rated on a 4-point Likert scale; six negatively worded items were reverse-coded. Total scores range from 37 to 148, with higher scores indicating better health conservation. Cronbach’s α was .94 in the original study and .81 in the present study.

### 4. Intervention

#### 1) Development of the health coaching program

A health coaching program for older adults with diabetes was developed based on Cox’s Interaction Model of Client Health Behavior. To clearly demonstrate the theoretical framework, the program components were explicitly mapped to the four key concepts of Cox’s interaction model: affective response, client–professional interaction, decisional control, and professional–technical competence. This alignment ensured that each session reflected the essential dimensions of client–provider interaction. A detailed composition of the program is presented in Table 1.

The intervention consisted of 16 sessions over 8 weeks, held twice weekly. Small-group sessions (7~8 participants) were adopted based on recent evidence demonstrating the effectiveness of group-based coaching and education in facilitating behavior change among older adults with chronic diseases [27].

To ensure content validity, a panel of nine experts—including nursing faculty, a physician, diabetes and gerontological nurse specialists, a physical education professor, and certified health coaches—evaluated the sessions using a 4-point Likert scale. The resulting Content Validity Index was .87, indicating strong content relevance.

#### 2) Implementation of the health coaching program

Based on pre-intervention HbA1c values, participants were divided into small groups of 7 to 8 individuals with similar glycemic control status. Those with poorer control, who were presumed to have greater difficulty with self-management, were grouped together to facilitate targeted support.

To enhance motivation and goal commitment, each group was assigned a specific HbA1c reduction goal. Participants also created team names and health slogans, which they recited at the beginning of each session to foster group identity and engagement. Educational content was delivered using a combination of multimedia tools such as PowerPoint slides, printed materials, and instructional videos.

Sessions were conducted in a small auditorium with chairs arranged for comfort and safety. Physical activities—including the "Happy dance," rhythmic movements to “Arirang,” stress-relief games, and stretching exercises—were carried out in the same space. To prevent accidents, seating was rearranged for activity sessions, and participants were instructed to wear appropriate clothing and footwear to reduce fall risk. Detailed session content is outlined in Table 2.

### 5. Data collection

The pretest was conducted immediately before the intervention for both the experimental and control groups. During the pretest, the researcher and trained research assistants administered structured questionnaires assessing general characteristics, self-care behaviors, diabetes distress, and health conservation. Physiological indicators, including HbA1c, FBG, TC, HDL-C, and blood pressure, were also measured at this time. Because some participants had visual or literacy difficulties, the research assistants read each questionnaire item aloud and recorded the participants’ responses. Each survey took approximately 25~30 minutes to complete. The posttest was administered immediately after the eight-week health coaching program using the same instruments as the pretest. HbA1c, FBG, TC, HDL-C, and blood pressure were remeasured to evaluate changes in physiological indicators.

### 6. Statistical analysis

All statistical analyses were performed using SPSS version 23.0 (IBM Corp., Armonk, NY, USA). Descriptive statistics were used to summarize the participants’ general characteristics and outcome variables. Categorical variables were presented as frequencies and percentages, whereas continuous variables were expressed as means and standard deviations.

To assess the homogeneity between the experimental and control groups, categorical variables were analyzed using the chi-square test or Fisher’s exact test, as appropriate, while continuous variables were examined using independent *t*-tests.

To evaluate the effectiveness of the intervention, change scores for each outcome variable were calculated, and between-group differences were analyzed using independent t-tests. Statistical significance was set at *p* < 0.05.

### 7. Ethical considerations

This study was approved by the Institutional Review Board (IRB) of Daegu Catholic University Hospital (IRB No. CR-16-094-L). All participants, including those in the control group, received sufficient explanation about the study purpose, procedures, anticipated benefits, and their rights before providing written informed consent. Participation was voluntary, and participants were assured of their right to withdraw at any time without penalty. The control group did not receive any intervention during the study period; they only underwent scheduled assessments, and their health data were used solely for research purposes. To address ethical considerations, health education materials were provided to the control group after the completion of the study. All collected data were kept confidential, and a small token of appreciation was provided to participants in both the experimental and control groups.

## RESULTS

### 1. General characteristics of participants

The participants in both groups were similar in general characteristics. The mean age was approximately 75 years, and the majority were women. Most participants were non-smokers, did not consume alcohol, and engaged in regular exercise. About half of the participants in both groups had been diagnosed with diabetes for more than 5 years (Table 3).

### 2. Homogeneity test between the experimental and control groups

No significant differences were found in the general characteristics between the experimental and control groups. In the pretest, physiological indicators (HbA1c, FBG, TC, HDL-C, and blood pressure) and psychosocial and behavioral variables (self-care behaviors, diabetes distress, and health conservation) also showed no significant differences between the groups, confirming that the groups were homogeneous before the intervention (Table 3).

### 3. Hypothesis testing

As shown in Table 4, the results of hypothesis testing were as follows.

**Hypothesis 1.** This hypothesis was partially supported, with five of the six physiological indicators showing significant improvement in the experimental group compared to the control group.

 **Sub-hypothesis 1-1.** HbA1c levels decreased significantly more in the experimental group than in the control group (t = 4.59, *p* < .001).

 **Sub-hypothesis 1-2.** FBG levels showed a significantly greater reduction in the experimental group (t = 6.66, *p* < .001).

 **Sub-hypothesis 1-3.** TC levels significantly decreased in the experimental group compared to the control group (t = 2.90, *p* = .005).

 **Sub-hypothesis 1-4.** No significant difference was found in HDL-C levels between the groups (t = 0.69, *p* = .492); therefore, this hypothesis was not supported.

 **Sub-hypothesis 1-5.** Systolic blood pressure showed a significantly greater reduction in the experimental group (t = 2.32, *p* = .024).

 **Sub-hypothesis 1-6.** Diastolic blood pressure also decreased significantly more in the experimental group (t = 2.94, *p* = .005).

**Hypothesis 2.** Self-care behavior scores increased significantly in the experimental group compared to the control group (t = 4.04, *p* < .001), supporting Hypothesis 2.

**Hypothesis 3.** Diabetes distress significantly decreased in the experimental group (t = 3.80, *p* < .001), supporting Hypothesis 3.

**Hypothesis 4.** Health conservation scores significantly improved in the experimental group (t = 3.20, *p* = .002), supporting Hypothesis 4.

## DISCUSSION

Diabetes mellitus is a prevalent chronic condition among older adults and is often accompanied by multiple comorbidities and functional limitations that complicate its management. Effective self-care behaviors and psychosocial well-being are essential for maintaining optimal glycemic control and preventing complications in this population. Guided by Cox’s interaction model, this study evaluated the effects of a health coaching program on physiological indicators, self-care behaviors, diabetes distress, and health conservation in older adults with diabetes. The findings provide empirical evidence to support nursing interventions that promote both physical and psychosocial health in this group.

Following the health coaching program, HbA1c in the experimental group decreased from 7.00% to 6.51%, whereas it increased from 6.92% to 7.14% in the control group, showing a significant between-group difference. This result is consistent with previous studies involving patients with type 2 diabetes [11,14], indicating that health coaching effectively reduces HbA1c levels. The improvement appeared to be related to the program’s focus on goal setting, self-directed behavior, and interactive engagement, which strengthened self-management capacity. Group coaching likely enhanced accountability, encouraged experience sharing, and supported individualized action plans [24]. In this study, participants with similar baseline HbA1c levels were grouped together, and weekly goals were established and monitored with peer feedback and professional coaching, which may have contributed to the favorable outcomes.

FBG significantly decreased in the experimental group (from 148.30 to 134.30 mg/dL) but increased in the control group (from 153.41 to 162.14 mg/dL), supporting the effectiveness of health coaching in lowering FBG levels [14,23]. Participants received glucometers and logbooks, were trained in self-monitoring techniques, and were encouraged to consistently track and manage their glucose, promoting self-regulation.

TC also significantly decreased in the experimental group, aligning with prior findings [14,24]. This reduction may be attributable to the combination of health consultations, dietary education, and exercise therapy. However, no significant change was observed in HDL-C, possibly due to the short intervention period and the limited frequency and intensity of exercise. In addition to glycemic and lipid improvements, blood pressure outcomes also showed meaningful change. Both systolic and diastolic blood pressure significantly decreased in the experimental group, consistent with previous studies targeting individuals with diabetes [14]. Regular blood pressure monitoring, stress-reduction activities, and motivation-focused coaching likely contributed to these improvements.

Self-care behaviors significantly improved in the experimental group, consistent with prior studies demonstrating the positive impact of nurse-led and peer-supported health coaching on self-care [14,28,29]. The program was tailored to the needs of older adults and included blood glucose monitoring education, case-based discussions, audiovisual materials, quizzes, and health pledge declarations to enhance motivation and responsibility for self-care. Beyond measurable physiological improvements, the program also enhanced psychosocial mechanisms such as patient–nurse interaction and self-efficacy, which are central to sustained self-management.

Diabetes distress also significantly decreased, consistent with earlier studies [9]. Stress was addressed through engaging physical activities using everyday items and thematic sessions with positive messaging (e.g., *“Making Friends with Diabetes”*). Peer support and mutual encouragement promoted emotional stability and sustained motivation. Additionally, health conservation improved significantly, reflecting not only better physical well-being but also integration of emotional, social, and functional dimensions. This improvement is likely due to enhanced self-care, reduced stress, and strengthened self-efficacy resulting from the health coaching program.

Grounding the intervention in Cox’s interaction model strengthened patient–nurse interaction, which is central to promoting self-management. Through structured dialogue, individualized goal setting, and continuous feedback, participants developed a sense of trust and partnership with nurses. This ongoing interaction provided both emotional and professional support, reinforcing self-efficacy and motivating participants to take greater initiative in managing blood glucose, maintaining lifestyle modifications, and coping with diabetes-related stress. Accordingly, the findings suggest that the model-based coaching approach not only facilitates behavioral change but also empowers older adults through relational support and confidence building.

A major strength of this study was its systematic design grounded in Cox’s interaction model, which provided a strong theoretical foundation for the intervention. The program effectively operationalized the model’s four core concepts: (1) affective response, reflected in laughter therapy and physical activities that fostered positive emotions; (2) client–professional interaction, reinforced through group coaching and continuous feedback from health professionals; (3) decisional control, supported by personalized goal setting and self-management planning; and (4) professional–technical competence, demonstrated through regular health monitoring and structured diabetes education. This integration of theory and practice established the program as a patient-centered nursing intervention that enhanced autonomy, motivation, and sustainable self-care among older adults with diabetes..

Despite these positive outcomes, the relatively short intervention period may have limited the depth of implementation for certain components. Furthermore, because the program incorporated multiple elements—such as education, stress management, group coaching, and health monitoring—it was difficult to isolate the specific contribution of each component. Future research should extend the intervention duration and adopt designs that enable component-level evaluation to clarify the mechanisms of effectiveness.

## CONCLUSION

This study evaluated the effects of a health coaching program based on Cox’s Interaction Model of Client Health Behavior on self-management in older adults with diabetes. The intervention resulted in significant improvements in physiological outcomes—including reductions in HbA1c, FBS, TC, and blood pressure—as well as psychosocial outcomes such as enhanced self-care behaviors, improved health conservation, and reduced diabetes distress. These findings demonstrate that a theory-based, client-centered coaching approach can effectively empower older adults with chronic illnesses to actively manage their health.

Overall, the results underscore the important role of nurses as facilitators of behavioral change through structured interaction, emotional support, and personalized goal setting. Incorporating nursing theory into community-based interventions reinforces the foundation of evidence-based practice and broadens the scope of preventive and promotive care.

Based on these findings, it is recommended to expand the application of Cox’s interaction model based health coaching to diverse populations and settings to verify its generalizability. Long-term follow-up studies are needed to assess the sustainability of the intervention’s effects, and future research should employ randomized controlled trial designs to provide stronger causal evidence for its effectiveness.

## Figures and Tables

**Table 1.** Mapping of Cox’s Interaction Model Concepts to Program Components

Cox’s interaction model concepts	Components of the health coaching program
Affective support	- Stress management sessions (laughter therapy, happy dance, arirang movements)
	- Sharing positive messages and team slogans
	- Incorporating games and fun elements for emotional support
Client–professional interaction	- Group coaching for goal setting and feedback
	- Regular health status assessment and individualized guidance
	- Sustained interaction between participants and professionals
Decision control	- Setting individual and team-specific glycemic control goals
	- Developing self-management plans (diet, exercise, medication)
	- Health pledge announcements and sharing achievements
Professional and technical competencies	- Health monitoring (blood pressure, blood glucose, cholesterol)
	- Diabetes education (dietary therapy, exercise, medication adherence)
	- Training in the use of glucometers and diabetes diaries

**Table 2.** Health Coaching Program with 16 Sessions

Week/session		Themes	Contents	Auxiliary materials	Interventions
1	1	Introduction to the program	∙Explaining how to progress the program	PPT	∙Health monitoring
			∙Introducing participants and determining nicknames	Handout	∙Health education
			∙Making health slogans	Video	
			∙Regulations of life for preventive management of cardiocerebrovascular diseases		
	2	Assessment of needs	∙Announcing team slogans	Handout	∙Group coaching
			∙Recognizing the state of blood sugar regulation	Sketchbook	
			∙Finding topics and strengths	Pens	
			∙Setting goals	Praise stickers	
2	3	Exercise therapy for diabetes	∙Preventive management of diabetic complications (exercise therapy)	PPT	∙Health education
			∙Importance of the exercise therapy	Handout	
			∙Cautions at the exercise therapy	Video	
			∙Stretching and walking exercise	Towels	
			∙Muscle strengthening exercise with household items	Water bottles	
	4	Diagnosis of health problems	∙Forming and maintaining coaching relations	Handout	∙Group coaching
			∙Exploring non-practice health behavior and its causes	Sketchbook	
			∙Diagnosing health problems	Pens	
			∙Making plans and inspiring self-confidence	Praise stickers	
			∙Making a self-management plan for diabetes		
3	5	Dietary therapy for diabetes	∙Preventive management of the diabetic complications (dietary therapy)	PPT	∙Health education
			∙Consuming a low-fat and not salty diet	Handout	
			∙Having cellulose and no smoking and drinking	Video	
			∙Calculating daily caloric requirement		
			∙Using the food substitution table		
	6	Setting priorities	∙Forming and maintaining coaching relations	Handout	∙Group coaching
			∙Exploring non-practiced health behaviors and causes	Sketchbook	
			∙Diagnosing health problems	Pens	
			∙Making plans and inspiring self-confidence	Praise stickers	
			∙Making a plan for self-management of diabetes		
4	7	Medication therapy for diabetes	∙Preventive management of diabetic complications (medication therapy)	PPT	∙Health education
			∙Importance of medication therapy	Handout	∙Health monitoring
			∙Cautions for medication therapy	Video	
			∙Self-monitoring of blood sugar	Diabetes diary	
			∙Keeping a diabetes diary	Glucose meter	
	8	Agreement on the goals of health management	∙Forming and maintaining coaching relations	Handout	∙Group coaching
			∙Exploring non-practiced health behaviors and causes	Sketchbook	
			∙Diagnosing health problems	Pens	
			∙Making plans and inspiring self-confidence	Praise stickers	
			∙Making a plan for self-management of diabetes		
5	9	Stress management	∙Managing the stress of diabetes (enjoying the twilight of their life)	PPT	∙Health education
			∙Sharing management of stress	Handout	
			∙How to cope with and solve stress	Video	∙Management of stress
	10	Checking the awareness of goals	∙Forming and maintaining coaching relations	Handout	∙Group coaching
			∙Exploring non-practiced health behaviors and causes	Sketchbook	
			∙Diagnosing health problems	Pens	
			∙Making plans and inspiring self-confidence	Praise stickers	
			∙Making a plan for self-management of diabetes		
6	11	Management of stress	∙Happy dance and arirang movements	PPT	∙Management of stress
			∙Stretching and laughter therapy	Video	
			∙Games for stress-relief	Balloons	
			∙The talent show competition between teams	Newspapers	
	12	Linkage of resources	∙Forming and maintaining coaching relations	Handout	∙Group coaching
			∙Exploring non-practiced health behaviors and causes	Sketchbook	
			∙Diagnosing health problems	Pens	
			∙Making plans and inspiring self-confidence	Praise stickers	
			∙Making a plan for self-management of diabetes		
7	13	Management of stress	∙Happy dance and arirang movements	PPT	∙Management of stress
			∙Stretching and games for stress-relief	Video	
			∙Having positive thoughts (accepting and studying diabetes)	Balloons	
			∙OX quiz: the golden bell of diabetes	Newspapers	
				Sketchbook	
	14	Setting and checking goals	∙Forming and maintaining coaching relations	Handout	∙Group coaching
			∙Exploring non-practiced health behavior and causes	Sketchbook	
			∙Diagnosing health problems	Pens	
			∙Making plans and inspiring self-confidence	Praise stickers	
			∙Making a plan for self-management of diabetes		
8	15	Management of stress	∙Health clapping and laughter therapy	Video	∙Management of stress
			∙Happy dance and arirang movements	Balloons	
			∙Holding a dance king contest	Newspapers	
	16	Assessment of goal achievement	∙Forming and maintaining coaching relations	Video	∙Group coaching
			∙Checking goal achievement	Sketchbook	∙Health monitoring
			∙Announcing the health pledge	Pens	
			∙Sharing participants’ opinions	Praise stickers	
			∙The award ceremony		

PPT = PowerPoint slides.

**Table 3.** Homogeneity Test of General Characteristics and Dependent Variables between Two Groups (N = 59)

Characteristics	Categories	Exp (n = 30)	Cont (n = 29)	χ^2^ (*p*)/t (*p*)
Sex	Men	12 (40.0)	11 (37.9)	0.03 (.871)
	Women	18 (60.0)	18 (62.1)	
Age (years)	65∼74	11 (36.7)	8 (27.6)	0.56 (.456)
	≥ 75	19 (63.3)	21 (72.4)	
		74.57 ± 3.27	75.17 ± 4.15	
Education	Below elementary school	9 (30.0)	9 (31.1)	1.26 (.532)
	Middle school	10 (33.3)	13 (44.8)	
	Above high school	11 (36.7)	7 (24.1)	
Living status	Alone	12 (40.0)	17 (58.6)	2.15 (.342)
	With spouse	13 (43.3)	8 (27.6)	
	With children	5 (16.7)	4 (13.8)	
Economic status	High	8 (26.7)	3 (10.3)	3.02 (.221)
	Moderate	20 (66.7)	2 2(75.9)	
	Low	2 (6.6)	4 (13.8)	
Smoking	Yes	2 (6.7)	5 (17.2)	1.58 (.254^‡^)
	No	28 (93.3)	24 (82.8)	
Alcohol	Yes	10 (33.3)	11 (37.9)	0.14 (.712)
	No	20 (66.7)	18 (62.1)	
Exercise	Yes	20 (66.7)	19 (65.5)	0.01 (.926)
	No	10 (33.3)	10 (34.5)	
Period of diagnosis (years)	＜ 5	12 (40.0)	14 (48.3)	0.41 (.522)
	≥ 5	18 (60.0)	15 (51.7)	
HbA1c (%)		7.00 ± 0.80	6.96 ± 1.15	0.14 (.893)
FBG (mg/dL)		148.30 ± 33.02	153.41 ± 28.25	0.64 (.526)
TC (mg/dL)		174.87 ± 50.38	173.52 ± 37.60	0.12 (.908)
HDL-C (mg/dL)		48.23 ± 11.23	46.10 ± 10.80	0.74 (.461)
SBP (mmHg)		143.50 ± 12.88	144.14 ± 16.37	0.17 (.868)
DBP (mmHg)		76.83 ± 9.05	78.10 ± 8.91	0.54 (.589)
Self-care behavior		71.40 ± 11.18	67.03 ± 12.15	1.44 (.156)
Diabetes distress		57.77 ± 11.32	57.93 ± 9.09	0.52 (.958)
Health conservation		105.87 ± 14.20	100.59 ± 10.36	0.70 (.490)

Values are presented as the mean ± standard deviation or n (%). Exp = Experimental group; Cont = Control group; HbA1c = Glycated hemoglobin; FBG = Fasting blood glucose; TC = Total cholesterol; HDL-C = High-density lipoprotein cholesterol. SBP = Systolic blood pressure; DBP = Diastolic blood pressure. ^‡^Fisher’s exact test.

**Table 4.** Changes in Dependent Variables in Both Groups (N = 59)

Variables	Group	Pre-test	Post-test	Diff	t (*p*)
HbA1c (%)	Exp (n = 30)	7.00 ± 0.80	6.51 ± 0.75	−0.49 ± 0.70	4.59 (< .001)
	Cont (n = 29)	6.96 ± 1.14	7.14 ± 0.99	0.18 ± 0.36	
FBG (mg/dL)	Exp	148.30 ± 33.02	134.30 ± 31.72	−14.00 ± 12.08	6.66 (< .001)
	Cont	153.41 ± 28.25	162.14 ± 30.42	8.72 ± 14.09	
TC (mg/dL)	Exp	174.87 ± 50.38	150.30 ± 27.86	−24.57 ± 32.00	2.90 (.005)
	Cont	173.52 ± 37.60	174.97 ± 37.91	1.45 ± 36.82	
HDL-C (mg/dL)	Exp	48.23 ± 11.23	46.87 ± 10.66	−1.37 ± 8.86	0.69 (.492)
	Cont	46.10 ± 10.80	47.48 ± 17.10	1.38 ± 19.82	
SBP (mmHg)	Exp	143.50 ± 12.88	138.50 ± 19.26	−5.00 ± 14.80	2.32 (.024)
	Cont	144.14 ± 16.37	146.76 ± 11.19	2.62 ± 9.84	
DBP (mmHg)	Exp	76.83 ± 9.05	74.17 ± 7.77	−2.67 ± 10.06	2.94 (.005)
	Cont	78.10 ± 8.91	82.28 ± 6.59	4.17 ± 7.62	
Self-care behavior	Exp	71.40 ± 11.18	83.30 ± 13.55	11.90 ± 14.63	4.04 (< .001)
	Cont	67.03 ± 12.15	63.21 ± 10.35	−3.83 ± 15.25	
Diabetes distress	Exp	57.77 ± 11.32	51.30 ± 9.20	−6.47 ± 6.92	3.80 (< .001)
	Cont	57.93 ± 9.09	60.34 ± 6.48	2.41 ± 10.69	
Health conservation	Exp	105.87 ± 14.20	114.10 ± 11.19	11.23 ± 15.04	3.20 (.002)
	Cont	100.59 ± 10.36	100.59 ± 11.19	0.00 ± 11.74	

Values are presented as the mean ± standard deviation. Exp = Experimental group; Cont = Control group; Diff = Mean difference; HbA1c = Glycated hemoglobin; FBG = Fasting blood glucose; TC = Total cholesterol; HDL-C = High-density lipoprotein cholesterol. SBP = Systolic blood pressure; DBP = Diastolic blood pressure.

## Data Availability

The data that support the findings of this study are available from the corresponding author upon reasonable request.
